# Design and Analysis of a High-Gain and Robust Multi-DOF Electro-thermally Actuated MEMS Gyroscope

**DOI:** 10.3390/mi9110577

**Published:** 2018-11-05

**Authors:** Muhammad Saqib, Muhammad Mubasher Saleem, Naveed Mazhar, Saif Ullah Awan, Umar Shahbaz Khan

**Affiliations:** 1 Department of Electrical Engineering, National University of Sciences and Technology, Islamabad 44000, Pakistan; naveed.mazhar@ceme.edu.pk (N.M.); saifullahawan@ceme.nust.edu.pk (S.U.A.); 2 Department of Mechatronics Engineering, National University of Sciences and Technology, Islamabad 44000, Pakistan; mubasher.saleem@ceme.nust.edu.pk (M.M.S.); u.shahbaz@ceme.nust.edu.pk (U.S.K.)

**Keywords:** MEMS gyroscope, electro-thermal actuator, non-resonant, multi-DOF, high gain, capacitive sensing, robustness

## Abstract

This paper presents the design and analysis of a multi degree of freedom (DOF) electro-thermally actuated non-resonant MEMS gyroscope with a 3-DOF drive mode and 1-DOF sense mode system. The 3-DOF drive mode system consists of three masses coupled together using suspension beams. The 1-DOF system consists of a single mass whose motion is decoupled from the drive mode using a decoupling frame. The gyroscope is designed to be operated in the flat region between the first two resonant peaks in drive mode, thus minimizing the effect of environmental and fabrication process variations on device performance. The high gain in the flat operational region is achieved by tuning the suspension beams stiffness. A detailed analytical model, considering the dynamics of both the electro-thermal actuator and multi-mass system, is developed. A parametric optimization is carried out, considering the microfabrication process constraints of the Metal Multi-User MEMS Processes (MetalMUMPs), to achieve high gain. The stiffness of suspension beams is optimized such that the sense mode resonant frequency lies in the flat region between the first two resonant peaks in the drive mode. The results acquired through the developed analytical model are verified with the help of 3D finite element method (FEM)-based simulations. The first three resonant frequencies in the drive mode are designed to be 2.51 kHz, 3.68 kHz, and 5.77 kHz, respectively. The sense mode resonant frequency is designed to be 3.13 kHz. At an actuation voltage of 0.2 V, the dynamically amplified drive mode gain in the sense mass is obtained to be 18.6 µm. With this gain, a capacitive change of 28.11 fF and 862.13 fF is achieved corresponding to the sense mode amplitude of 0.15 μm and 4.5 μm at atmospheric air pressure and in a vacuum, respectively.

## 1. Introduction

Accelerometers and gyroscopes are the most dominant among the MEMS-based inertial sensors. The unprecedented and remarkable success of inertial sensors in consumer and portable electronics, especially in smart phones and gaming consoles, has made MEMS gyroscopes the expeditiously growing sector in the MEMS inertial sensors industry. MEMS-based inertial sensors have a wider application spectrum spanning from advanced automotive safety systems and short range navigation and guidance systems for unmanned aerial vehicles (UAVs) to interactive consumer electronics including gaming consoles, mobile phones, and image stabilization in cameras [1,2,3,4,5]. The conventional high-performance fiber optic, ring laser, and rotating wheel based gyroscopes are costly and have a large size which makes them incompatible for most of the potential emerging applications. The MEMS gyroscopes offer a better alternative owing to their miniaturized size, low cost, and possible integration with integrated circuit (IC) technology [6]. MEMS gyroscopes consist of a vibrating mechanical element for sensing angular velocity. The working principle is based on the transfer of energy between the drive and sense modes as a result of Coriolis acceleration corresponding to input angular velocity. In the drive mode, the vibrating mass is oscillated at a constant amplitude whereas in the sense mode the orthogonal oscillation of sense mass due to input angular velocity is measured [7,8]. The drive mode actuation and sense mode oscillation detection in MEMS gyroscopes is generally carried out using electrostatic transduction mechanisms due to their low-input power requirement [9,10,11]. However, the output displacement of electrostatic actuators is low. The electrothermal actuation mechanism, based on the Joule heating effect, offers relatively large output force and displacement at low actuation voltage and have small footprint [12,13,14]. 

The conventional MEMS gyroscopes have a mechanical structure with 1-DOF drive and sense mode. These gyroscopes are termed as “resonant gyroscopes” in which high mechanical gain and sensitivity is achieved by tuning the resonant frequencies of two modes to match. However, the resonant frequency matching becomes extremely difficult due to microfabrication process tolerances and environmental parametric variations. Moreover, the coupling mechanisms between two modes and corresponding quadrature error is also of major concern in resonant MEMS gyroscopes [15,16,17,18]. To overcome the mode matching issue in resonant MEMS gyroscopes, multi-DOF gyroscope designs have been presented in literature [9,19,20,21,22,23,24,25]. The DOF in either drive or sense mode are increased to achieve relatively wide operating bandwidth for increased robustness. The gyroscopes are operated in the flat region created between the two resonant peaks, thus avoiding the need for strict frequency matching between drive and sense modes. These multi-DOF gyroscopes are termed as “non-resonant” MEMS gyroscopes.

The major problem faced by non-resonant gyroscopes is the intrinsically lower response outside the vicinity of the resonant peak positions. To address this issue, several different approaches are adopted in literature. An earlier solution for this issue was proposed using the concept of micro machined coupled resonators with dynamic vibration absorber structure [26]. The concept of dynamic motion amplification seems to be a feasible solution to the stated problem. In this approach, the basic two mass oscillator controls the distance between two resonance peaks based on the mass ratios which is a non-viable way of controlling the mechanical amplification based on the fact that second mass needs to be very small in order to achieve large mechanical amplification. This problem is solved by improved two mass system in which an added spring structure reduced the dependency on the mass ratios for mechanical amplification, but this approach is still insufficient to provide adequate control to endure imperfections due to the drift of springs with temperature variations and fabrication imperfections [27]. A new three-mass coupled resonator is proposed to resolve the issue with improved two mass system [28]. In this three-mass coupled resonator, the resonance and anti-resonance positions can be easily regulated by changing the spring constant rather than changing the whole structure. Moreover, the mechanical amplification in three-mass coupled resonators is less affected by mass ratios and can be further improved by altering the spring constants which is more practical way to enhance the dynamic amplification.

In this paper, a 4-DOF MEMS gyroscope is proposed based on the concept of three-mass coupled resonators with a 3-DOF drive mode and 1-DOF sense mode. The gyroscope is designed by considering the microfabrication process constraints of commercially available low-cost process MetalMUMPs. [29]. This dynamic system is composed of three masses constituting of a 3-DOF drive mode for dynamic amplification in the drive mode while the third mass being mechanically decoupled from the drive mode form the 1-DOF sense mode. The drive and sense mode oscillations are mechanically decoupled from each other with the help of decoupling frame. The frequency response of 3-DOF drive mode oscillator shows that the dynamic system has three resonance peaks with flat regions in between the peaks. The sense mode resonant frequency is designed to lie between the flat region of first and second resonance peak where the response gain is least sensitive to environmental changes and parametric fluctuations.

## 2. Operating Principle of the Proposed Gyroscope

**Figure 1:**
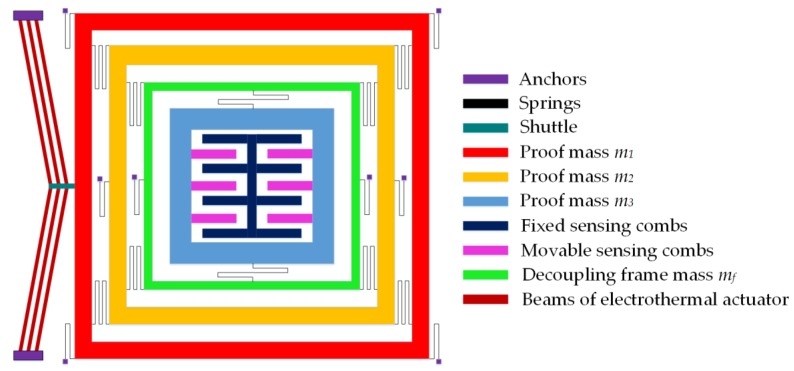
Schematic diagram of the proposed 4-DOF MEMS gyroscope with a 3-DOF drive mode and 1-DOF sense mode oscillator.

Figure 1 shows the schematic of the proposed MEMS gyroscope with a 3-DOF drive mode and 1-DOF sense mode. The first mass $m_{1}$ which is the drive mass is excited by an external force in the drive direction using the V-shaped electro thermal actuator. The second mass $m_{2}$ transfers the dynamic energy from the mass $m_{1}$ to mass $m_{3}$ which acts as a final oscillating mass. The masses $m_{1}$ and $m_{2}$ are free to move in the drive direction but constrained to move in the sense direction by means of elastic springs. The sense mode oscillations of mass $m_{3}$ are decoupled from the drive direction oscillations by means of a decoupling frame $m_{f}$ to minimize instabilities due to dynamical coupling between sense and drive modes. Thus, masses $m_{1}$, $m_{2}$, and combination of $\left(m_{f}+m_{3}\right)$ form a 3-DOF drive mode while $m_{3}$ being free to oscillate in the sense acts as a 1-DOF sense mode oscillator. The sense direction oscillation of the mass $m_{3},$ corresponding to an input angular velocity, is designed to be measured by using parallel plate sense combs.

## 3. Analytical Modeling for the Proposed MEMS Gyroscope

### 3.1. Dynamic Equations for Drive and Sense Mode

The equations of motion for the proposed MEMS gyroscope are derived using the Lagrange equation, given as [30]

(1) $$\frac{d}{dt}\left(\frac{\partial K}{\partial {\dot{x}}_{i}}\right)-\frac{\partial K}{\partial x_{i}}+\frac{\partial D}{\partial {\dot{x}}_{i}}+\frac{\partial V}{\partial x_{i}}=f$$

where *K* is kinetic energy, *V* is potential energy, *D* is dissipation function, and *f* is the forcing function of the vibrating system. The subscript *i* depicts the degree of freedom.

**Figure 2:**
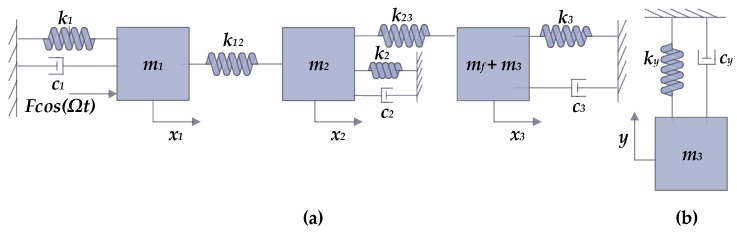
Lumped mass-spring-damper model of the proposed gyroscope, (**a**) 3-DOF drive mode, (**b**) 1-DOF sense mode.

Figure 2 shows the lumped mass-spring-damper model for the proposed microgyroscope. The kinetic energy, potential energy, and dissipative function for this model can be written as

(2) $$K=\frac{1}{2}m_{1}{\dot{x}}_{1}^{2}+\frac{1}{2}m_{2}{\dot{x}}_{2}^{2}+\frac{1}{2}\left(m_{f}+m_{3}\right){\dot{x}}_{3}^{2}+\frac{1}{2}m_{3}{\dot{y}}_{1}^{2}$$

(3) $$V=\frac{1}{2}k_{1}x_{1}^{2}+\frac{1}{2}k_{12}{\left(x_{1}-x_{2}\right)}^{2}+\frac{1}{2}k_{2}x_{2}^{2}+\frac{1}{2}k_{23}{\left(x_{2}-x_{3}\right)}^{2}+\frac{1}{2}k_{3}x_{3}^{2}+\frac{1}{2}k_{y}y_{1}^{2}$$

(4) $$D=\frac{1}{2}c_{1}{\dot{x}}_{1}^{2}+\frac{1}{2}c_{2}{\dot{x}}_{2}^{2}+\frac{1}{2}c_{3}{\dot{x}}_{3}^{2}+\frac{1}{2}c_{y}{\dot{y}}_{1}^{2}$$

By employing the Lagrange equation to Equations (2)–(4), the 4-DOF system of equations are derived as

(5) $$m_{1}{\ddot{x}}_{1}+c_{1}{\dot{x}}_{1}+k_{1}x_{1}+k_{12}\left(x_{1}-x_{2}\right)=F\cos\left(\Omega t\right)$$

(6) $$m_{2}{\ddot{x}}_{2}+c_{2}{\dot{x}}_{2}+k_{2}x_{2}+k_{12}\left(x_{2}-x_{1}\right)+k_{23}\left(x_{2}-x_{3}\right)=0$$

(7) $$\left(m_{3}+m_{f}\right){\ddot{x}}_{3}+c_{3}{\dot{x}}_{3}+k_{3}x_{3}+k_{23}\left(x_{3}-x_{2}\right)=0$$

(8) $$m_{3}{\ddot{y}}_{1}+c_{y}{\dot{y}}_{3}+k_{y}y_{3}=0$$

For frequency analysis, the above equations can be transformed into a matrix form given as

(9) $$\left[M\right]\ddot{X}+\left[C\right]\dot{X}+\left[K\right]X=\left[F_{D}\right]$$

where matrices *M, C* and *K* are given as

(10) $$\left[X\right]=\left[\begin{array}{c} x_{1}\\ x_{2}\\ x_{3}\\ y_{1} \end{array}\right]$$

(11) $$\left[M\right]=\left[\begin{array}{cccc} m_{1} & 0 & 0 & 0\\ 0 & m_{2} & 0 & 0\\ 0 & 0 & m_{3}+m_{f} & 0\\ 0 & 0 & 0 & m_{3} \end{array}\right]$$

(12) $$\left[C\right]=\left[\begin{array}{cccc} c_{1} & 0 & 0 & 0\\ 0 & c_{2} & 0 & 0\\ 0 & 0 & c_{3} & 0\\ 0 & 0 & 0 & c_{y} \end{array}\right]$$

(13) $$\left[K\right]=\left[\begin{array}{cccc} k_{1}+k_{12} & -k_{12} & 0 & 0\\ -k_{12} & k_{2}+k_{12}+k_{23} & -k_{23} & 0\\ 0 & -k_{23} & k_{3}+k_{23} & 0\\ 0 & 0 & 0 & k_{y} \end{array}\right]$$

If the system of equation has solution

(14) [X]=[A]sin(ωt)+[B]cos(ωt)

where *A* and *B* are vectors.

(15) $$\left[K\right]-\omega^{2}\left[M\right]=\left[\begin{array}{cccc} k_{1}+k_{12}-\omega^{2}m_{1} & -k_{12} & 0 & 0\\ -k_{12} & k_{2}+k_{12}+k_{23}-\omega^{2}m_{2} & -k_{23} & 0\\ 0 & -k_{23} & k_{3}+k_{23}-\omega^{2}\left(m_{f}+m_{3}\right) & 0\\ 0 & 0 & 0 & k_{y}-\omega^{2}m_{3} \end{array}\right]$$

(16) $$\omega \left[C\right]=\left[\begin{array}{cccc} \omega c_{1} & 0 & 0 & 0\\ 0 & \omega c_{2} & 0 & 0\\ 0 & 0 & \omega c_{3} & 0\\ 0 & 0 & 0 & \omega c_{y} \end{array}\right]$$

(17) $$\left[T\right]=\left[\begin{array}{cc} \left[K\right]-\omega^{2}\left[M\right] & -\omega \left[C\right]\\ \omega \left[C\right] & \left[K\right]-\omega^{2}\left[M\right] \end{array}\right]$$

The relation among *A, B* and *T* is given by

(18) $$\left[\begin{array}{c} \left[A\right]\\ \left[B\right] \end{array}\right]={\left[T\right]}^{-1}\left[\begin{array}{c} \left[F\right]\\ \left[0\right] \end{array}\right]$$

Once A and B are known, the frequency response can be obtained as

(19) $$x_{i}\left(\omega \right)=\sqrt{A_{i}+B_{i}}$$

### 3.2. Mechanical Stiffness and Damping Analysis

**Figure 3:**
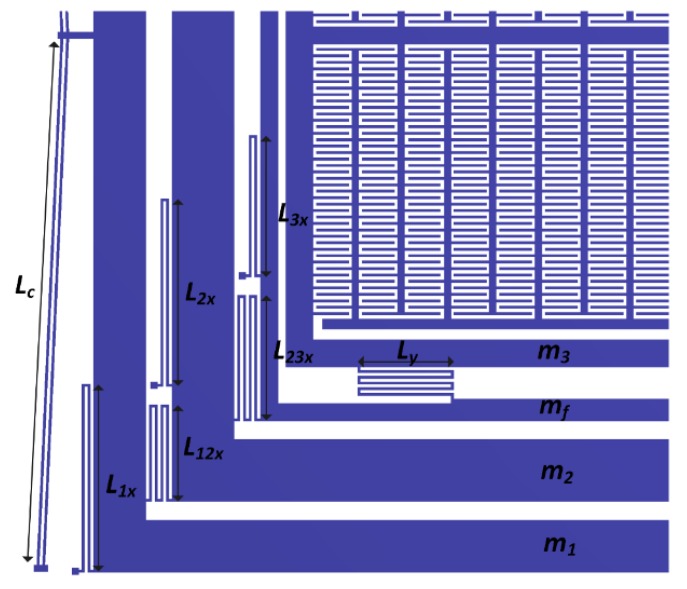
Drive and sense mode flexural suspension for the proposed MEMS gyroscope.

The designed suspension system configuration of the proposed gyroscope allows the masses $m_{1}$ and $m_{2}$ to move only in the drive direction but restricted their motion in the sense direction. The mass $m_{3}$ is allowed to oscillate in both the drive and sense direction which are orthogonal to each other. The suspension system configuration of the proposed gyroscope dynamical model is shown in Figure 3.

The four, double folded flexures connect the masses $m_{1}$, $m_{2}$, and $m_{3}$ with the substrate through anchors with each having beams of length $L_{1x}$, $L_{2x}$ and $L_{3x}$, respectively. These folded flexures beam can be modeled as a fixed-guided beams, resulting in an overall stiffness of

(20) $$k_{1}=\frac{4}{2}\left(\frac{12EI}{L_{1x}^{3}}\right)=\frac{2Etw^{3}}{L_{1x}^{3}}$$

(21) $$k_{2}=\frac{4}{2}\left(\frac{12EI}{L_{2x}^{3}}\right)=\frac{2Etw^{3}}{L_{2x}^{3}}$$

(22) $$k_{3}=\frac{4}{2}\left(\frac{12EI}{L_{3x}^{3}}\right)=\frac{2Etw^{3}}{L_{3x}^{3}}$$

where w, L, and t are the width, length, and thickness of the beams, respectively, E is the Young’s Modulus of the material and $I=tw^{3}/12$ is the second moment of inertia of the rectangular beam. The stiffness of chevron-shaped electro thermal actuator being directly connected to the mass $m_{1}$, significantly affects the natural frequency of the proposed dynamical system in the drive direction. The chevron shaped actuator can be modeled as a fixed-fixed micro beam by neglecting the small inclination angle θ. The stiffness $k_{chev}$ of the chevron-shaped actuator consisting of N beams, each having length $L_{c}$, can be calculated as [31]

(23) $$k_{chev}=N\left(\frac{192EI}{L_{c}^{3}}\right)=N\left[\frac{16Etw^{3}}{L_{c}^{3}}\right]$$

The overall drive direction stiffness of mass $m_{1}$ can be estimated by merging $k_{1x}$ and $k_{chev}$ as

(24) $$k_{1eq}=\left[\frac{2Etw^{3}}{L_{1x}^{3}}\right]+N\left[\frac{16Etw^{3}}{L_{c}^{3}}\right]$$

The mass $m_{1}$ is connected to mass $m_{2}$ which is then connected to the decoupling frame having mass $m_{f}$ via four drive direction deformable quadruple-folded flexures having lengths $L_{12x}$ and $L_{23x}$ and their stiffness is given by $k_{12}$ and $k_{23}$, respectively.

(25) $$k_{12}=\frac{4}{1}\left(\frac{12EI}{L_{12x}^{3}}\right)=\frac{4Etw^{3}}{L_{12x}^{3}}$$

(26) $$k_{23}=\frac{4}{1}\left(\frac{12EI}{L_{23x}^{3}}\right)=\frac{4Etw^{3}}{L_{23x}^{3}}$$

In order to minimize the zero rate drift and dynamic coupling between the drive and sense directions of the proposed gyroscope dynamical model, the sense mass $m_{3}$ is coupled to the decoupling frame via four sense direction quintuple-folded flexures, with all the beams of length $L_{3y}$. The overall sense direction stiffness $k_{3y}$ of the sense mass is

(27) $$k_{3y}=\frac{4}{5}\left(\frac{12EI}{L_{3y}^{3}}\right)=\frac{4Etw^{3}}{5L_{3y}^{3}}$$

The fluidic friction due to air between the sliding surfaces of the proof masses and the stationary surface of the substrate is the major energy dissipation mechanism in the vibratory MEMS gyroscopes. The total slide film damping $c_{1x}$ and $c_{2x}$ for the mass $m_{1}$ and $m_{2}$ oscillating in the drive direction can be demonstrated as Couette flow by considering an instantaneously established linear fluidic velocity profile and is given as

(28) $$c_{1}=\mu_{e}\frac{A_{1}}{z_{0}}$$

(29) $$c_{2}=\mu_{e}\frac{A_{2}}{z_{0}}$$

The total slide film damping $c_{3x}$ for the combination of masses $m_{3}+\;m_{f}$ oscillating in the drive direction can be calculated by assuming the Couette flow between the stationary substrate and the proof mass along with considering Couette flow between the sensing combs and is given as:

(30) $$c_{3}=\mu_{e}\frac{\left(A_{3}+A_{f}\right)}{z_{0}}+\mu_{e}\frac{2N_{cap}l_{cap}t}{y_{cap}}$$

The total damping $c_{3y}$ for the mass $m_{3}$ oscillating in the sense direction results from the Couette flow between the stationary substrate and the proof mass along with the squeeze film damping between the sensing combs and is given as

(31) $$c_{y}=\mu_{e}\frac{A_{3}}{z_{0}}+\mu_{e}\frac{7N_{cap}l_{cap}t^{3}}{y_{cap}^{3}}$$

where $\mu_{e}$ is the effective permittivity of the air, A is the area of the sliding surface, $z_{0}$ is the distance between the proof mass and the stationary substrate, $N_{cap}$ is the no of air gap capacitors, $l_{cap},t$ and $y_{cap}$ are the overlapping length, thickness, and distance moved by the sensing combs respectively.

### 3.3. Output Force and Displacement Analysis of Electrothermal Actuator

**Figure 4:**
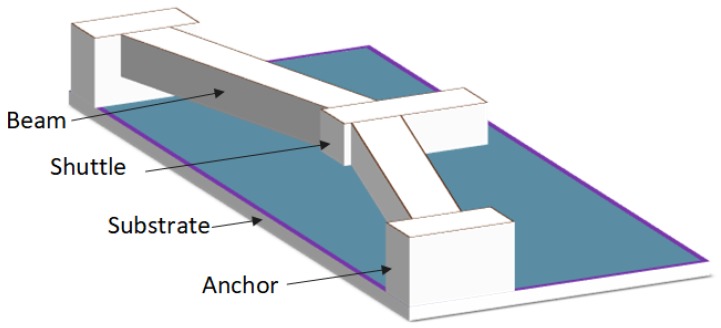
Schematic diagram of the proposed chevron-shaped electro-thermal actuator.

The schematic of V-shaped electro thermal micro actuator is shown in Figure 4. Heat is generated due to Joule heating on the application of a potential difference *V* on the anchors of the beams, which results into forward pushing of the central shuttle due to the symmetrical expansion of the V-shaped beams.

The output displacement of the chevron-shaped electro-thermal actuator in the closed-form, by combining the electric-thermal and thermo-mechanical models, can be written as [32]

(32) $$y_{max}=\left(F_{1}-F_{2}\right)\left(\begin{array}{c} V^{2}\\ P_{E} \end{array}\right)$$

where, $F_{1}$ and $F_{2}$ are the flexibilities of the thermal actuator due to voltage V and external force $P_{E}$ respectively. Corresponding stiffness are: K1=1F1 and Kchev=1F2. The voltage and external forces are similar in terms of causing tip displacement and generating force. Equation (32) can be rewritten as

(33) $$y_{max}=F_{2}.F_{max}=F_{2}.\left(P_{V}-P_{E}\right)$$

where PV=(F1F2)V2 shows the corresponding force for the applied voltage. $F_{max}$ is the output force generated by the electrothermal actuator having stiffness $K_{chev}$ at the maximum displacement $y_{max}$ and is given by

(34) $$F_{max}=\left(P_{V}-P_{E}\right)$$

Assuming ζ=F1F2, the output force of electrothermal actuator can be written as

(35) $$F_{max}=\zeta V^{2}-P_{E}$$

The impacts of the input parameters (i.e., voltage and external load), structural parameters (i.e., beams and shuttle geometry), material properties (i.e., coefficient of thermal expansion, modulus of elasticity and resistivity), on the output responses (i.e., maximum force output and tip displacement) can be obtained using Equations (33) and (35).

**Figure 5:**
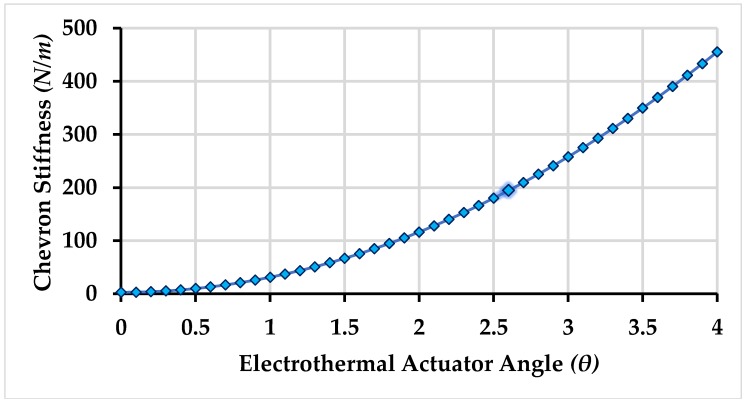
Stiffness values of the electro-thermal actuator with angle variations.

**Figure 6:**
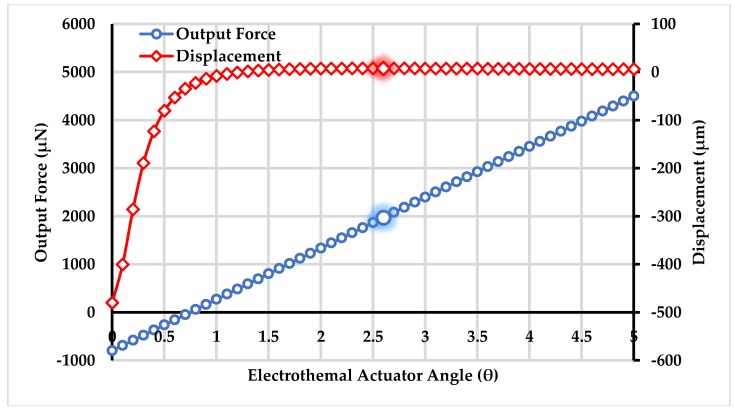
Force output and maximum displacement values of the electro-thermal actuator with angle variations.

The stiffness-force model of the electro-thermal actuator shows a relationship between output force and displacement by considering the actuator to be a spring with stiffness $K_{chev}$. The stiffness of the actuator being dependent on the inclination angle θ plays an imperative role in the response of the proposed non-resonant gyroscope. Figure 5 and Figure 6 shows the impact of the inclination angle θ on the stiffness, output force generation, and displacement, respectively. The inclination angle θ is chosen to be 2.6° based on the fact that maximum displacement of 7.396 μm took place at that angle with reasonable amount of output force 1971.4 μN and stiffness value of 194.47 N/m in the presence of an external load.

**Figure 7:**
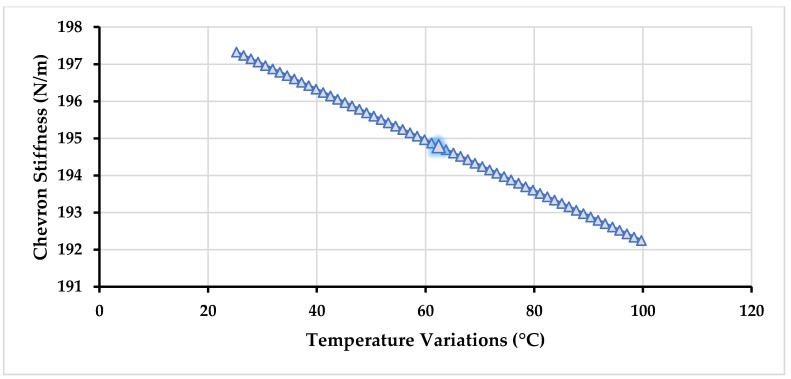
Stiffness values of the electro-thermal actuator with temperature variations.

There is also a minute dependence of electrothermal actuator stiffness on the temperature as shown in Figure 7 and there is small change in the stiffness of the chevron-shaped electro-thermal if the temperature rises as the result of applied input voltage due to the decrease in the value of Young’s modulus of nickel [33]. In our proposed gyroscope, the highest temperature is 62.3 °C on the arms of the chevron-shaped actuator and the corresponding stiffness is 194.47 N/m.

## 4. Parametric Design Optimization of the Proposed MEMS Gyroscope

**Figure 8:**
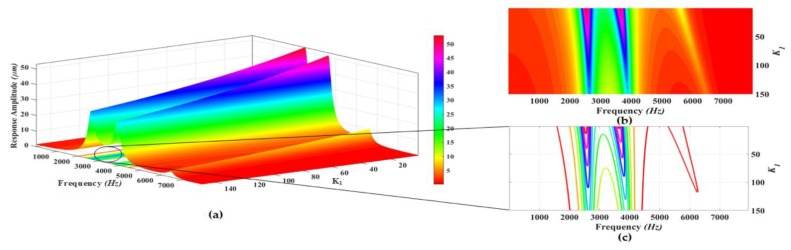
(**a**) Response amplitude and bandwidth optimization graph for the variation of $k_{1}$; (**b**) Top view and (**c**) Contour plots of the system response.

**Figure 9:**
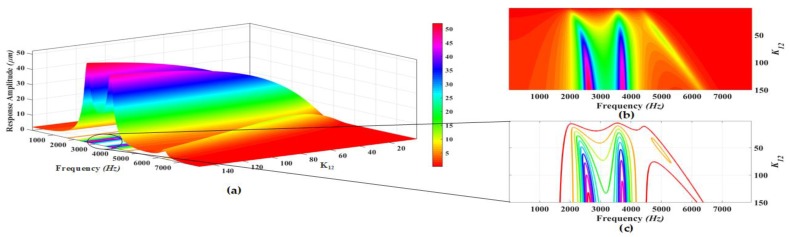
(**a**) Response amplitude and bandwidth optimization graph for the variation of $k_{12}$; (**b**) Top view and (**c**) Contour plots of the system response.

**Figure 10:**
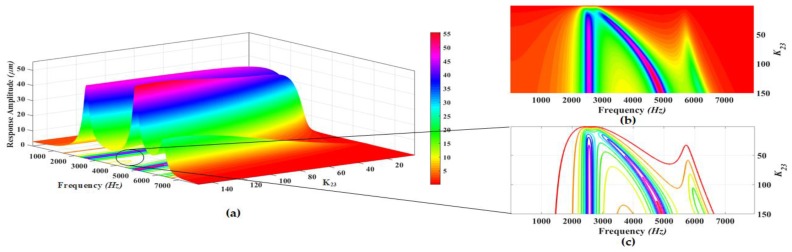
(**a**) Response amplitude and bandwidth optimization graph for the variation of $k_{23}$; (**b**) Top view and (**c**) Contour plots of the system response.

**Figure 11:**
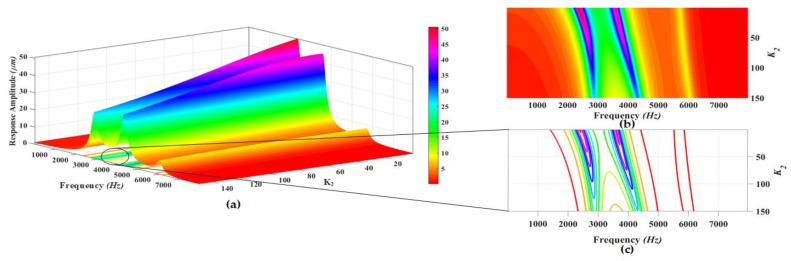
(**a**) Response amplitude and bandwidth optimization graph for the variation of $k_{2}$; (**b**) Top view and (**c**) Contour plots of the system response.

**Figure 12:**
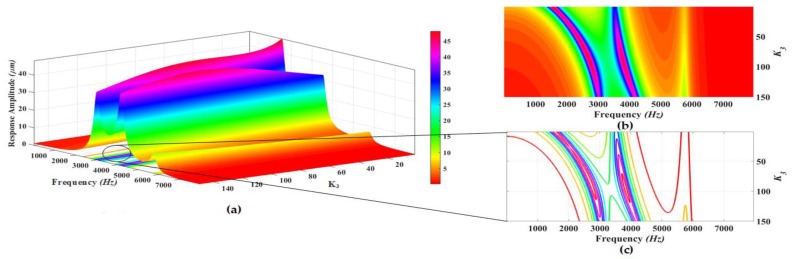
(**a**) Response amplitude and bandwidth optimization graph for the variation of $k_{3}$; (**b**) Top view and (**c**) Contour plots of the system response.

The proposed MEMS gyroscope is designed to be operated in region between the first two drive mode resonance peaks which allows to minimize the effect of environmental variations and microfabrication process uncertainties. Thus, to maximize the gain in the flat operational region between first two resonant peaks, a parametric design optimization is carried out by varying the stiffness values of different suspensions connecting the masses to each other and to substrate. The parametric optimization of the proposed gyroscope dynamical model is done by maximizing the concerned variables response using grid search method [34,35]. In this method, the maxima of curves are explored for certain realizable range of values for the parameters being optimized. The 3D graphs are plotted using analytical model and the system response including gain in the region between resonance peaks and positions of resonance peaks is analyzed for varying stiffness values. The analysis is carried out at a fixed actuation voltage of 0.2 V to electrothermal actuator. Figure 8, Figure 9, Figure 10, Figure 11 and Figure 12 show the system response and operational range for the proposed gyroscope dynamical model in the drive mode by changing the stiffness values of the mechanical springs including $k_{1}$, $k_{2}$, $k_{3}$, $k_{12}$, and $k_{23}$.

Figure 8a shows the response amplitude and bandwidth graph of the proposed electro-thermally actuated microgyroscope for the variation of $k_{1}$. Figure 8b, c shows the top view and contour plots of the system response and bandwidth, respectively. The response amplitude of the system is shown by the legend in Figure 8. The dynamic response of the system shows that position of the first, second, and third resonance frequencies moves to higher values by increasing the value of the $k_{1}$. But the position of the third resonance peak is most significantly affected by changing the value of $k_{1}$. The response amplitudes have the highest values at the resonance peaks for lower values of $k_{1}$. But with the increase in the value of $k_{1}$, the response amplitudes show a decreasing trend on the resonance peaks. Also the value of the response amplitude in the valley between the first and second resonant frequency shows a decreasing trend with increasing the value of $k_{1}$ as shown by the colors legend in the top view of the system response.

Figure 9a shows the response amplitude and bandwidth graph of the proposed electro-thermally actuated microgyroscope dynamic model for the variation of $k_{12}$. Figure 9b,c shows the top view and contour plots of the system response and bandwidth, respectively. The response amplitude of the system is shown by the legend in the Figure 9. The dynamic response of the system shows that position of first and third resonance frequencies moves to higher values while the position of the second resonance frequency moves to lower value by increasing the value of the $k_{12}$. But the position of third resonance peak is significantly affected by changing the value of $k_{12}$. The response amplitudes have higher values at the first and second resonance peaks while for the third resonance peak the response amplitude has lower values for higher values of $k_{12}$. But with the increase in the value of $k_{12}$, the response amplitudes show the increasing trend on the first and second resonance peaks and a decreasing trend for the third resonance peak. Also the value of the response amplitude in the valley between the first and second resonant frequency shows the increasing trend with increasing the value of $k_{12}$ as shown by colors of legend in the top view of the system response.

Figure 10a shows the response amplitude and bandwidth graph of the proposed electro-thermally actuated microgyroscope dynamic model for the variation of $k_{23}$. Figure 10b,c shows the top view and contour plots of the system response and bandwidth, respectively. The response amplitude of the system is shown by the legend in Figure 10. The dynamic response of the system shows that the position of first resonance frequency moves to a lower value while the positions of the second and third resonance frequencies moves to higher values by increasing the value of the $k_{23}$. But the position of the second resonance peak is most significantly affected by changing the value of $k_{23}$ and at a very low value of $k_{23}$, the third resonance peak almost dies out. The response amplitudes have the highest values at the resonance peaks for higher values of $k_{23}$. With the increase in the value of $k_{23}$, the response amplitudes show a decreasing trend on the resonance peaks, but the response amplitude on the first resonance frequency is least affected by the variation in $k_{23}$ except at $k_{23}=0$ at which it dies out. Also the value of the response amplitude in the valley between the first and second resonant frequency shows a decreasing trend with increasing the value of $k_{23}$ as shown by colors of legend in the top view of the system response.

Figure 11a shows the response amplitude and bandwidth graph of the proposed electro-thermally actuated microgyroscope dynamic model for the variation of $k_{2}$. Figure 11b,c shows the top view and contour plots of the system response and bandwidth, respectively. The response amplitude of the system is shown by the legend in the Figure 11. The dynamic response of the system shows that position of first and second resonance frequencies moves to higher values by increasing the value of the $k_{2}$. But the position of third resonance peak is least affected by changing the value of $k_{2}$. The response amplitudes have the highest values at the resonance peaks for lower values of $k_{2}$. But with the increase in the value of $k_{2}$, the response amplitudes show a decreasing trend on the resonance peaks. Also the value of the response amplitude in the valley between the first and second resonant frequency shows a decreasing trend with increasing the value of $k_{2}$ as shown by colors of the legend in the top view of the system response.

Figure 12a shows the response amplitude and bandwidth graph of the proposed electro-thermally actuated microgyroscope dynamic model for the variation of $k_{3}$. Figure 12b,c shows the top view and contour plots of the system response and bandwidth, respectively. The response amplitude of the system is shown by the legend in the Figure 12. The dynamic response of the system shows that position of first and second resonance frequencies moves to higher values while the position of third resonance frequency moves to lower value by increasing the value of the $k_{3}$. But the position of first resonance peak is most affected by changing the value of $k_{3}$. The response amplitude has the highest value at the first resonance peak for lower values of $k_{3}$. But with the increase in the value of $k_{3}$, the response amplitudes show a decreasing trend except at values near $k_{3}=100$ on the first resonance peak. Also the response amplitude shows the increasing trend till $k_{3}=100$ on the second resonance peak after which it again starts decreasing. The value of the response amplitude in the valley between the first and second resonant frequency shows an increasing trend with increasing the value of $k_{3}$ till at $k_{3}=100$ after which it also starts decreasing as shown by the colors legend in the top view of the system response. The optimized design parameters for the drive mode oscillator obtained through 3D graphs are listed in the Table 1.

## 5. FEM-Based Electro-Thermal-Structural Analysis

**Figure 13:**
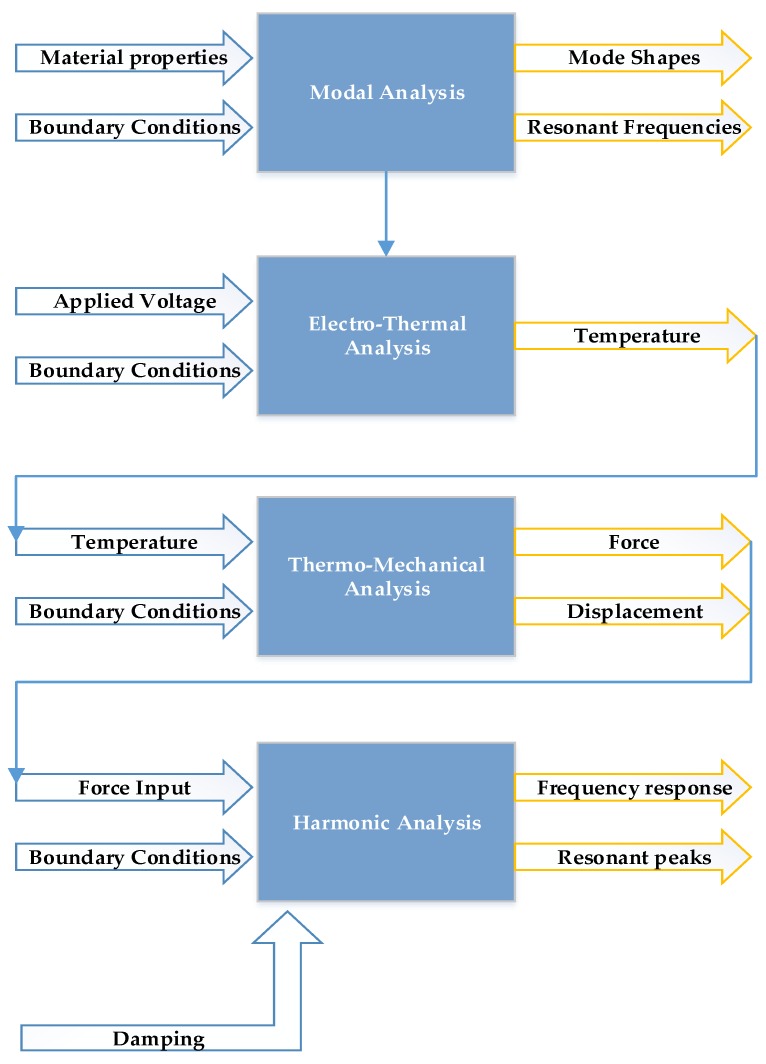
The sequential diagram for the FEM-based analysis.

In this section, a detailed FEM-based simulation methodology for the proposed microgyroscope is presented. Starting with the modal analysis, the different mode shapes and resonant frequencies of the structure are shown. Initially, a static structural analysis is performed to analysis the temperature distribution, output force, and displacement of the electro-thermal actuator. Afterwards, a modal and harmonic analysis is performed to analyze the frequency response of the dynamic system for 3-DOF drive mode oscillator. The material properties used for the thin-film nickel in the FEM simulations are summarized in Table 2. Figure 13 shows the sequential diagram for the FEM-based analysis in which blue arrows indicate the input parameters while the yellow arrows indicate the output response of the specific analysis.

### 5.1. Modal Analysis

**Figure 14:**
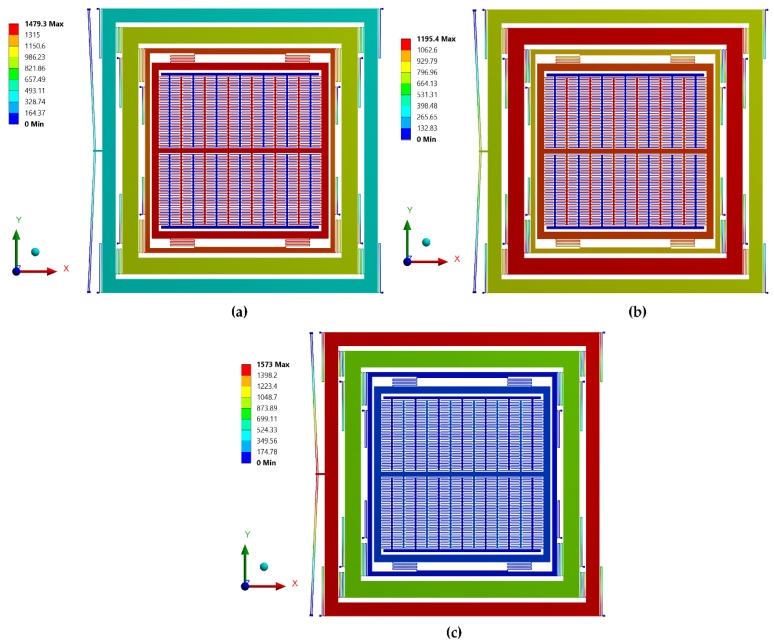
Combined modal analysis results of the overall 4-DOF proposed gyroscope dynamical model. (**a**) Mode shape at 1st drive mode resonant frequency. (**b**) Mode shape at 2nd drive mode resonant frequency. (**c**) Mode shape at 3rd drive mode resonant frequency.

To verify the natural frequency response using the developed analytical model in Section 3.1, a FEM-based natural frequency analysis is carried out. The boundary conditions and material properties are input parameters while mode shapes and resonant frequencies are the output responses of the modal analysis. The structural parts are modeled using Solid 98 elements. The optimized mesh size is selected based on patch conforming algorithm for tetrahedrons method control. The desired mode shapes with their associated resonant frequencies are shown in Figure 14. Table 3 shows the comparison of the drive mode resonant frequencies obtained using FEM analysis to that obtained using an analytical model. The percentage deviation is less than 1%, thus validating the accuracy of the developed analytical model.

### 5.2. Electro-Thermal and Thermal Structural Analysis for Actuator

**Figure 15:**
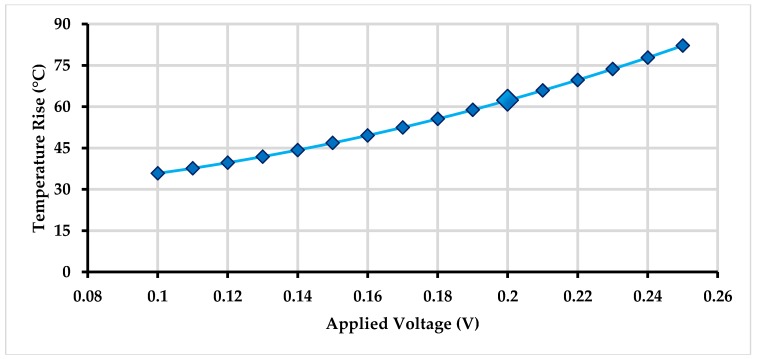
Temperature rise values of the electro-thermal actuator with applied voltage variations.

**Figure 16:**
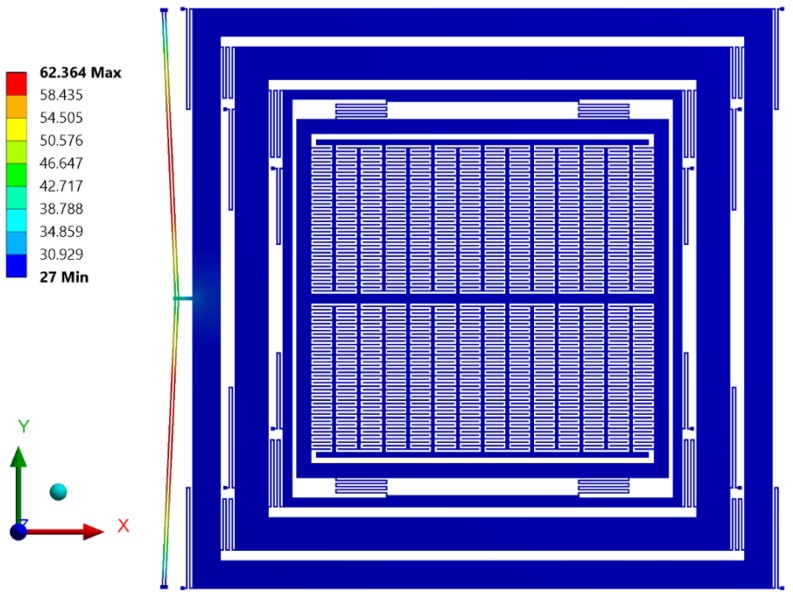
Temperature profile in the proposed MEMS gyroscope.

Initially, an electro-thermal analysis was carried out to predict the temperature rise in the electro-thermal actuator from ambient for an applied input static actuation voltage. Figure 15 shows the electro-thermal actuator temperature rise with increasing input actuation voltage. For an actuation voltage up to 0.25 V, the maximum temperature in the electro-thermal actuator was less than 100 °C. Figure 16 shows the temperature distribution profile in the proposed MEMS gyroscope. The maximum temperature was concentrated in the electro-thermal actuator beams while the temperature value in the masses of the gyroscope was negligible.

**Figure 17:**
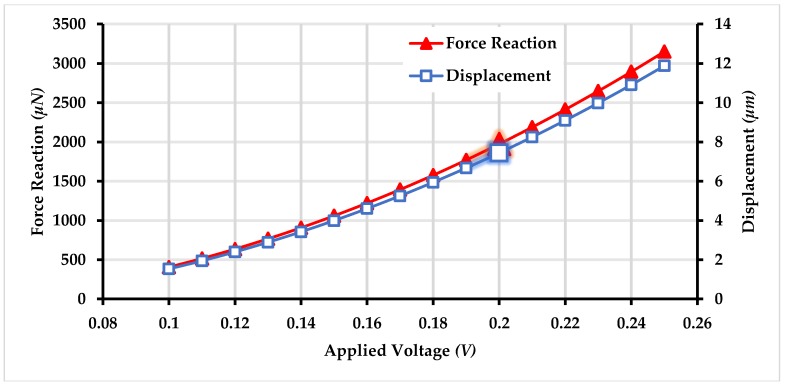
Output force and displacement of the electro-thermal actuator with increasing actuation voltage.

**Figure 18:**
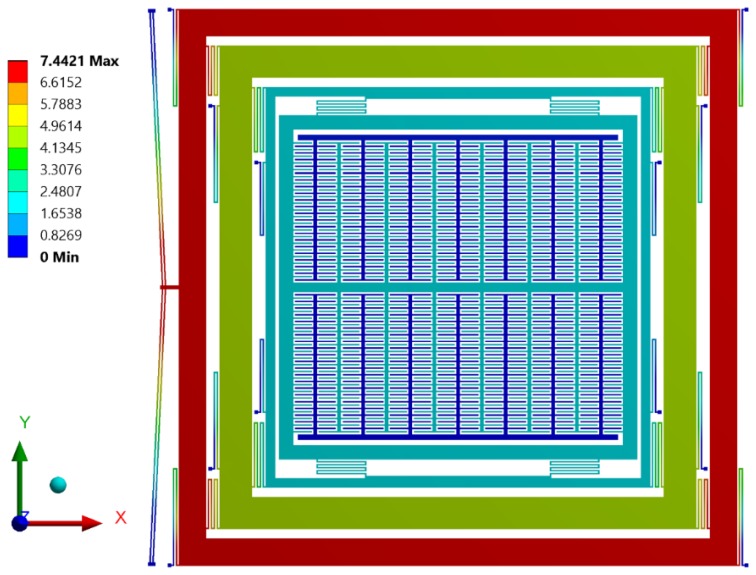
Thermo-mechanical analysis showing the maximum displacement achieved by the device at 0.2 V DC.

Based on the temperature values obtained using electro-thermal analysis for an applied actuation voltage, a FEM-based thermo-structural analysis was carried out to analyze the output force generated and displacement produced by the chevron-shaped electro-thermal actuator. Figure 17 shows the output displacement and force generated by the actuator for the actuation voltages of up to 0.25 *V*. The results show that with increasing actuation voltage both the output force and displacement are in the range of micro Newton and micrometer respectively. Figure 18 shows the displacement corresponding to the applied voltage of 0.2 *V*.

### 5.3. Frequency Response Analysis 

**Figure 19:**
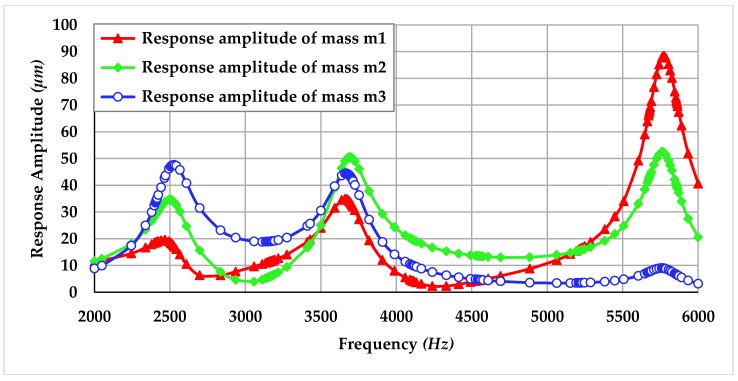
Frequency response of the proposed MEMS gyroscope in the drive mode.

The harmonic analysis was carried out to analyze the frequency response of the proposed MEMS gyroscope. The force reaction of the electrothermal actuator obtained through thermal-structural analysis corresponding to an actuation voltage of 0.2 *V* was used to perform the harmonic analysis. The response amplitude of masses was directly dependent on the air pressure and corresponding air damping. The values of damping incorporated in the harmonic response analysis were obtained by the relations presented in Section 3.2. Figure 19 shows the frequency response of three masses $m_{1}$, $m_{2}$ and $m_{3}$ in the drive mode obtained through FEM simulations.

The result shows that at first resonant frequency of 2.51 kHz, the displacement in the masses $m_{1}$, $m_{2}$ and $m_{3}$ is 20 µm, 35 µm and 48 µm respectively. The dynamic displacement amplification in mass $m_{3}$ is 2.4 times with respect to mass $m_{1}$. At second resonant frequency of 3.68 kHz, the dynamic displacement in mass $m_{2}$ is highest as compared to mass $m_{1}$ and $m_{3}$. At third resonant frequency of 5.8 kHz, the mass $m_{1}$ moves with highest amplitude while the displacement in the sense mass $m_{3}$ is only 8 µm. The dynamically amplified gain for the sense mass $m_{3}$ between the first two resonant peaks is 18.6 µm while the gain between second and third resonant peak is negligible. Thus, flat operational region between first two resonant peaks with bandwidth of nearly 1.2 kHz is the most suitable region for the operation of the proposed non-resonant MEMS gyroscope.

**Figure 20:**
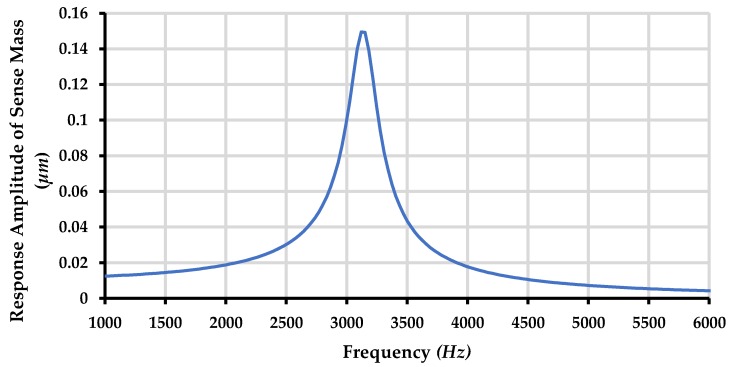
Response amplitude of the sense mass $m_{3}$ of the proposed MEMS gyroscope for ambient conditions.

**Figure 21:**
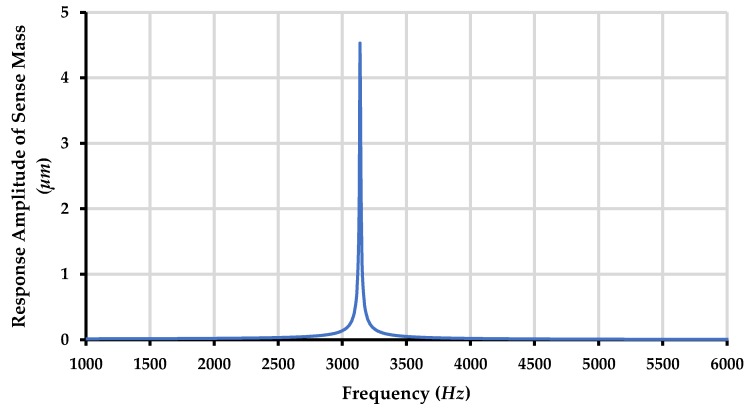
Response amplitude of the sense mass $m_{3}$ of the proposed MEMS gyroscope for vacuum conditions.

The frequency response of the sense mass m_3_ is shown in Figure 20 and Figure 21 respectively for both the ambient and vacuum conditions which shows that that sense mode resonant frequency lies in the middle of first two resonant frequencies at 3.13 kHz.

**Figure 22:**
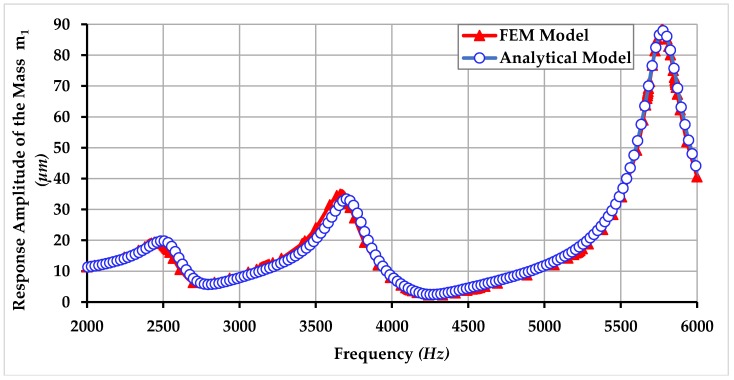
Comparison of response amplitude of the mass $m_{1}$ of the proposed MEMS gyroscope obtained using FEM and an analytical model.

**Figure 23:**
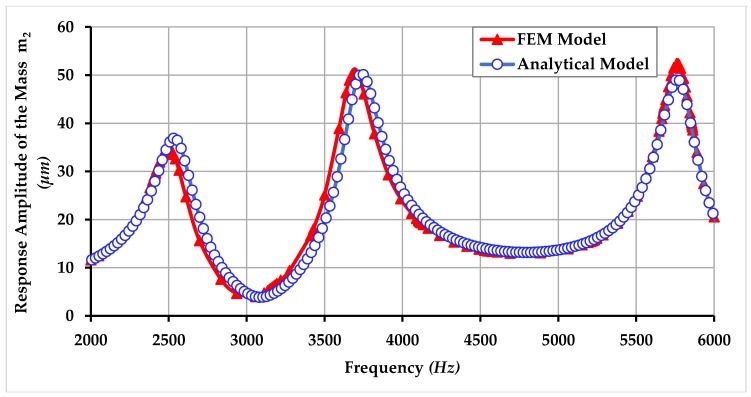
Comparison of response amplitude of the mass $m_{2}$ of the proposed MEMS gyroscope obtained using FEM and an analytical model.

**Figure 24:**
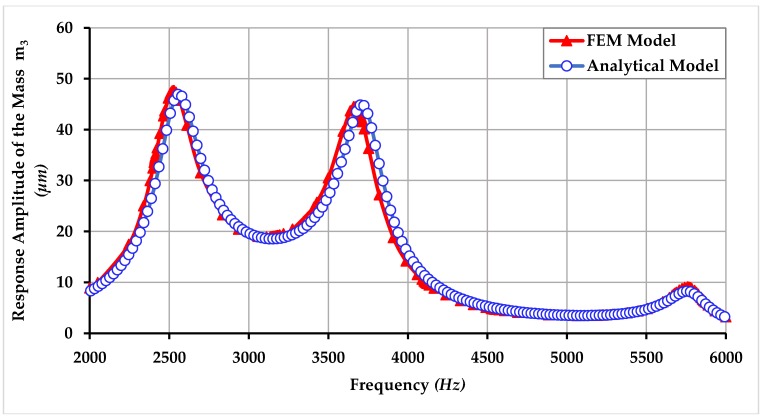
Comparison of response amplitude of the mass $m_{3}$ of the proposed MEMS gyroscope obtained using FEM and an analytical model.

In this section, the comparison of harmonic analysis using FEM analysis and analytical modeling is also presented and it shows that the analytical simulation results lie in good accordance with the FEM analysis results. Comparison of response amplitude of the masses $m_{1}$, $m_{2}$, and $m_{3}$ of the proposed gyroscope dynamical model using FEM and analytical modeling is shown in Figure 22, Figure 23 and Figure 24. The small difference between the resonance peaks and response amplitude is due to the small difference between the values of spring constants obtained analytically and obtained through FEM-based simulations as shown in the Table 1.

### 5.4. Robustness Analysis

**Figure 25:**
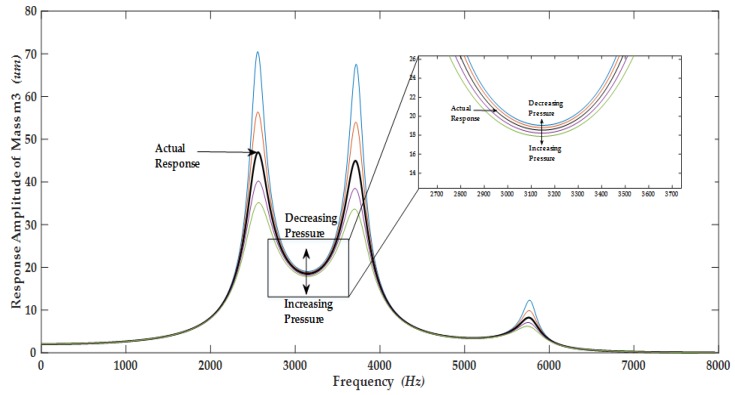
Response amplitude of mass m_3_ showing the robustness of the proposed gyroscope against the environmental changes and fluctuations.

Table 4 shows the parametric fluctuations which could occur due to the limitation of the microfabrication process MetalMUMPs. The actual values of gain and new values of gain as a result of expected changes in the nominal values of the different structural parameters are also shown in the Table 5, which depicts that the proposed gyroscope has the potential to overcome the fabrication imperfections. Figure 25 shows the robustness of the proposed gyroscope against the environmental fluctuations, as there is the minor effect of environmental fluctuations on the gain of the proposed gyroscope dynamic response in the flat region between the first two resonant peaks.

### 5.5. Sensitivity Analysis

The sensitivity of MEMS gyroscope is dependent on the Coriolis force Fc = 2m*Ω*v where *m* is the sense mass, *Ω* is the angular rotation to be measured and *v* is the velocity of the sense mass in the drive direction. For the resonant gyroscopes, since the mass is oscillated at resonance frequency the displacement amplitude of sense mass is much higher and hence induced Coriolis force is also high. However, achieving precise resonance is very challenging at the microscale owing to both fabrication process uncertainties and temperature variations. For the proposed non-resonant MEMS gyroscope, the sense mass *m_3_* was oscillated in the drive direction by using an electro-thermal actuator, and the Coriolis force corresponding to input rotation was designed to be measured by using a parallel plate actuator. The sense mass *m_3_* was optimized to be operated at a frequency of 3.13 k Hz which lies in the middle of region between the first two drive mode resonance peaks of the MEMS gyroscope. This minimizes the effect of external conditions. Moreover, the suspension beams of the sense mass in the y-direction are optimized in such a way that the sense mode resonance frequency matches the operational frequency of 3.13 kHz.

**Figure 26:**
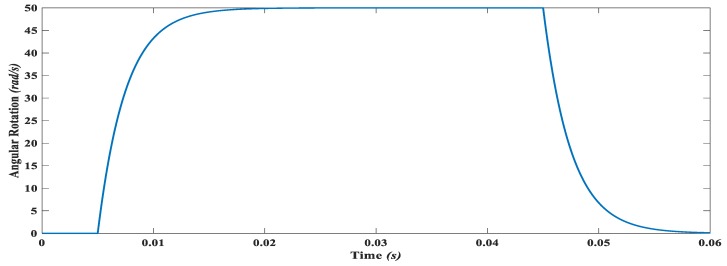
Angular rotation about z-axis applied to the proposed MEMS gyroscope.

**Figure 27:**
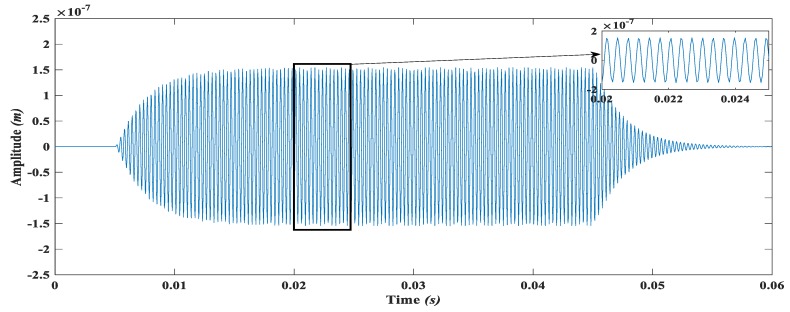
Sense mass displacement amplitude in the y-axis due to induced Coriolis force.

The input angular velocity of 50 rad/s. is applied to the proposed gyroscope as shown in Figure 26 and the response of the gyroscope is observed and shown in Figure 27. With the input angular velocity of the 50 rad/s and the drive direction amplitude of 18.6 μm of the mass $m_{3}$, the displacement produced in the sense direction due to rotation impelled Coriolis force is 0.15 μm. The sense direction displacement of the mass $m_{3}$. is calculated with the help of sense combs in terms of capacitance change ΔC. by using the formula given as

(36) $$\Delta C=2N\varepsilon_{0}tly/y_{0}^{2}.$$

where *N* is the total no of the sense direction parallel plate capacitors, t is the thickness of the vibrating structure, l is the overlapping length of the comb fingures, $y_{0}$ is the initial separation in the sensing combs and y is the sense direction displacement of the mass $m_{3}$ as a result of rotation impelled Coriolis force. The capacitance change measured through Equation (36) is 28.11 fF. If the same device is operated at vacuum, the value of the measured capacitance change is increased and is measured to be 862.13 fF for the same angular rate of 50 rad/s due to the fact that decreasing the pressure inside the encapsulated device package results in low energy dissipation due to both slide and squeeze film damping phenomena [37]. The results of the capacitance change show that the sensitivity of the device can be improved by 10 manifolds by operating the device in vacuum. The sensitivity of our device is 0.565 fF/rad/s for atmospheric while 17.24 fF/rad/s for in vacuum conditions.

## 6. Discussion

Electro-thermal actuators are better in performance to electrostatic interdigitated comb drive actuators in terms of generating large force and output displacements at lower actuation voltages and small actuator foot prints. Moreover, the metal-based electro-thermal actuators have an added advantage of a large thermal expansion coefficient which results in even larger output displacements and force generation than their Silicon-based counterparts. The lateral deflection of the silicon-based chevron-shaped electro-thermal actuators is almost 60% less than that of the nickel metal-based electro-thermal actuators, under the comparable power consumption [38]. Thermal time constant plays a significant role in the overall dynamic response of the system, as the operational frequency of the electro-thermally actuated microgyroscope is governed by the heating and cooling rate of the electro-thermal actuators arms corresponding to the applied voltage. Lower thermal time constant leads to overall faster heating and cooling rates. The metals have better thermal time constant in comparison to silicon and polysilicon and among the different metals electroplated nickel has the best material properties to be used as heat actuators [38]. Moreover, insufficient time for heating and cooling results in buildup of temperature on the device which might result in the potential failure of the device [39,40,41]. Thus, for the reliable actuation using electro-thermal actuators, the resonant frequency is kept below 5 kHz. So our gyroscope was designed keeping in view the requirement of feasible heating and cooling rates.

The mass spring system proposed by Erismis [28] allows to have dynamic displacement amplification in the sense mass of the proposed gyroscope in the flat valley present between the first two resonance peaks. The advantages of the 3-DOF drive mode over 2-DOF counterpart is in terms of high gain. Moreover, the design can be altered more effectively by only changing the most significant spring constants instead of changing the whole structure in the 3-DOF drive mode oscillator to achieve desired bandwidth without significantly sacrificing the gain. Furthermore, this approach leads to robust structure which has the improved tendency to encounter the fabrication imperfections and environmental fluctuations as compared to the 2-DOF counterpart. Additionally, the drift in the spring due to environmental changes can be more easily tolerated by using this structural approach.

The environmental temperature plays a vital role in the performance of the device. Young’s modulus of the nickel tends to decrease with increasing temperature, and the increase in temperature tend to change the resonant frequencies being directly related to Young’s modulus of the electroplated nickel [33]. Moreover, temperature fluctuations significantly affect the resonator’s quality factor [42]. At high temperature, quality factor of the proposed gyroscope will be reduced [1,43]. But as the proposed gyroscope is made to operate in the dynamic valley created between the first two resonance frequencies, the impact of quality factor variations has the minimum effect on the performance of the device as can be seen in Figure 25.

High gain and insensitivity of the proposed gyroscope to the structural fabrication imperfections and environmental changes made that gyroscope a potential candidate for the reliable, robust, and highly-sensitive gyroscope. Moreover, in this paper, the impact of inclination angle on force output and displacement generation and stiffness of the chevron-shaped actuator is clearly depicted which previously was approximated by using the simple relation for stiffness calculation by neglecting the bent angle of the chevron-shaped arms, which is rather an inappropriate way of estimating the stiffness of the chevron-shaped actuator. The choice of the proper actuator angle leads to overall greater force and displacement generation for driving the gyroscope at a reasonably low power consumption. Furthermore, this work gives the insight on the impact of the changing spring constants on gain and bandwidth simultaneously using the aid of 3D graphs. This approach facilitates the designer to design the application specific gyroscope by considering the most significant design parameter.

The power consumption of the single hot arm of the chevron-shaped actuator is given by

(37) $$P=\frac{V^{2}}{R}=\frac{V^{2}A}{\rho L}$$

where V is the applied voltage, A is the area, ρ is the resistivity, and L is the anchor to anchor length of the electro-thermal actuator. Since in our gyroscope, the chevron-shaped electro-thermal actuator consists of two hot arms, therefore the power consumption of the proposed electro-thermally actuated gyroscope was obtained by multiplying the Equation (37) by two. The power consumption by our proposed design is *22 mW* which is significantly less in comparison to other actuation methods. Additionally, it is far less than the electro-thermal gyroscopes proposed in the literature.

Moreover, due to the stiffness values, the air damping strongly affects the sense mass displacement amplitude. Since MEMS gyroscopes are generally operated in a high-quality factor environment using specialized packaging, the sensitivity analysis of the proposed gyroscope both in atmospheric air pressure and vacuum conditions is carried out. At an actuation voltage of 0.2 V, the dynamically amplified drive mode gain in the sense mass was obtained as 18.6 µm. With this gain and angular rotation of 50 rad/s in the z-axis, a capacitive change of 28.11 fF for a displacement of 0.15 µm in the sense mass at atmospheric air pressure was achieved. However, by assuming vacuum environment, the displacement amplitude of sense mass increases to 4.5 µm with corresponding capacitance change of 862.13 fF in sense parallel plates. A comprehensive comparison of the proposed 4-DOF MEMS gyroscope with the already developed multi-DOF MEMS gyroscopes is presented in Table 6.

## 7. Conclusions

In this paper, a design implementation and verification of a high-gain electro-thermally actuated 3-DOF drive mode and 1-DOF sense mode vibratory MEMS gyroscope is presented. The performance parameters of the proposed gyroscope are analyzed and optimized by developing a detailed analytical model. The gyroscope design parameters are optimized using process constraints of low-cost commercially available micro fabrication process MetalMUMPs. The results obtained through an analytical model are verified through 3D FEM simulations. A high gain of 18.6 μm was achieved in the non-resonant flat operational region of the proposed gyroscope by utilizing 3-DOF drive model oscillator at a relatively lower input actuation voltage of 0.2 V. With this gain, a capacitive change of 28.11 fF and 862.13 fF was achieved at atmospheric air pressure and in vacuum, respectively. The proposed MEMS gyroscope allows to achieve high gain in the non-resonant MEMS gyroscopes which eventually leads to higher sensitivity in addition to robustness against the environmental and fabrication process variations.

## Figures and Tables

**Table 1:** The summary of the optimized design parameters for drive mode oscillator.

Parameters	Analytical Values
$m_{1}$	3.0036 × 10^−7^ kg
$m_{2}$	3.00 × 10^−7^ kg
$m_{f}$	7.9276 × 10^−8^ kg
$m_{3}$	2.3178 × 10^−7^ kg
$m_{f}+m_{3}$	3.1864 × 10^−7^ kg
$k_{chev}$	194.47 N·m^−1^
$k_{1x}=\frac{2Etw^{3}}{L_{1x}^{3}}$	30 N·m^−1^
$k_{2x}=\frac{2Etw^{3}}{L_{2x}^{3}}$	30 N·m^−1^
$k_{3x}=\frac{2Etw^{3}}{L_{3x}^{3}}$	70 N·m^−1^
$k_{12x}=\frac{2Etw^{3}}{L_{12x}^{3}}$	110 N·m^−1^
$k_{23x}=\frac{2Etw^{3}}{L_{23x}^{3}}$	50 N·m^−1^

**Table 2:** The material properties used for thin-film Nickel in FEM based simulations [36].

Property	Value	Unit
Thermal conductivity	91	W·m^−1^·K^−1^
Air conductivity	0.0256	W·m^−1^·K^−1^
Coefficient of thermal expansion	12.7 × 10^−6^	K^−1^
Electrical resistivity	200 × 10^−9^	Ω·m
Young’s modulus	214 × 10^9^	N·m^−2^
Poisson’s ratio	0.3	--
Density	8908	kg·m^−3^

**Table 3:** Comparison of the analytical and FEM-based modal analysis results.

Resonant Frequencies	FEM Analysis (*Hz*)	Analytical Model (*Hz*)	Percentage Deviation
1st drive mode resonant frequency	2518.1	2552.5	0.898%
2nd drive mode resonant frequency	3680.4	3725.5	0.012%
3rd drive mode resonant frequency	5772.1	5775.4	0.0005%

**Table 4:** Different parameter perturbations which could affect the robustness of the proposed gyroscope.

Parameter	Nominal Size (µm)	Expected Change (µm)
$w_{1x}$	8	±0.5
$w_{12x}$	8	±0.5
$w_{23x}$_._	8	±0.5
$w_{2x}$	8	±0.5
$w_{3x}$	8	±0.5
$L_{1x}$	526.6	±0.5
$L_{12x}$	271.1	±0.5
$L_{23x}$_._	352.6	±0.5
$L_{2x}$	526.6	±0.5
$L_{3x}$	397.1	±0.5

**Table 5:** Influence of parameter perturbation on the robustness of the prosed gyroscope.

Factors	Gain
Actual Value	18.54 µm
For (−0.5 um) change in parameters	18.42 µm
Percentage Deviation	0.65%
For (+0.5 um) change in parameters	18.49 µm
Percentage Deviation	0.27%

**Table 6:** Comparison of the proposed gyroscope with multi-DOF microgyroscopes presented in the literature.

Reference	Actuation Method	Sensing Method	DOF	Size	Gain in Drive Mode	Mechanical Sensitivity/
Shakoor et al. [44]	Electro-thermal	Capacitive	2-DOF Drive and 1-DOF Sense Mode	2.2 × 2.6 mm^2^	0.9 nm	1.7 μm/°/s
Saleem et al. [17]	Electrostatic	Capacitive	2-DOF Drive and 1-DOF Sense Mode	2.4 × 1.6 mm^2^	0.2µm	0.045 fF/rad/s
Riaz et al. [10]	Electrostatic	Capacitive	2-DOF Drive and 1-DOF Sense Mode	2.2 × 2.6 mm^2^	0.3 µm	…
Acar et al. [45]	Electrostatic	Capacitive	2-DOF Drive and 2-DOF Sense Mode	…	5 µm	0.72 × 10^−3^ μm/°/s
This work	Electro-thermal	Capacitive	3-DOF Drive and 1-DOF Sense Mode	3.2 × 3 mm^2^	18.6 µm	0.565 fF/rad/s
